# Momentum for policy change: alternative explanations for the increased interest in results-based financing in Uganda

**DOI:** 10.1080/16549716.2021.1948672

**Published:** 2021-07-30

**Authors:** Freddie Ssengooba, Aloysius Ssennyonjo, Timothy Musila, Elizabeth Ekirapa-Kiracho

**Affiliations:** a School of Public Health, Makerere University; b Ministry of Health, Kampala Uganda, Hanoi, Uganda

**Keywords:** Policy change, result-based financing, policy deliberations, Uganda

## Abstract

**Background:**

Results-based financing initiatives have been implemented in many countries as stand-alone projects but with little integration into national health systems. Results-based financing became more prominent in Uganda’s health policy agenda in 2014–2015 in the context of the policy imperative to finance universal health coverage.

**Objective:**

To explore plausible explanations for the increased policy interest in the scale-up of results-based financing in Uganda.

**Methods:**

In this qualitative study, information was collected through key informant interviews, consultative meetings (2014 and 2015) and document reviews about agenda-setting processes. The conceptual framework for the analysis was derived from the work of Sabatier, Kingdon and Stone.

**Results:**

Four alternative policy arguments can explain the scale-up of results-based financing in Uganda. They are: 1) external funding opportunities tied to results-based financing create incentives for adopting policies and plans; 2) increased expertise by Ministry of Health officials in the implementation of results-based financing schemes helps frame capacity accumulation arguments; 3) the national ownership argument is supported by increased desire for alignment and fit between results-based financing structures and legitimate institutions that manage the health system; and 4) the health systems argument is backed by evidence of the levers and constraints needed for sustainable performance. Shortages in medicines and workforce are key examples. Overall, the external funding argument was the most compelling.

**Conclusion:**

The different explanations illustrate the strengths and the vulnerability of the results-based financing policy agenda in Uganda. In the short term, donor aid has been the main factor shifting the policy agenda in favour of results-based financing. The high cost of results-based financing is likely to slow implementation. If results-based financing is to find a good fit within the Ugandan health system, and other similar settings, then policy and action are needed to improve system readiness.

## Background

During the last two decades, results-based financing (RBF) was promoted as a way of enhancing the performance of health systems. RBF refers to ‘a cash payment or non-monetary transfer made to a national or sub-national government, manager, provider, payer or consumer of health services after attainment and verification of predefined results’ [1]. It has been argued that RBF can be a ‘powerful means of increasing the quality and quantity of health services by way of promoting a results-orientation – linking incentives to desired outputs and encouraging entrepreneurial behaviours by staff and managers’ [2–4].

Proponents of RBF assert that RBF can increase the volume and quality of health services, reduce the costs of service provision, enhance effectiveness and efficiency, and improve staff motivation and retention [2–4]. However, critics express caution with regard to adopting RBF. They draw attention to the paucity of evidence for RBF scale-up, the embedded complexities in healthcare delivery, cost pressures, and unlikely sustainability in low-resource settings [5–7]. While the successful integration of RBF into national health systems has occurred in a few countries like Cambodia, Armenia and Rwanda [2,8,9], most have not moved beyond pilots and demonstration projects [5–7,10]. In their study, Ssennyonjo et al [1]. explored RBF pilots, study designs and healthcare objectives in the context of Uganda’s health system over the past two decades. The authors observe that ongoing re-design and evolving capacity development were necessary factors for RBF to become embedded and sustained in Uganda.

Like many other fundamental reforms in health financing, the scale-up of RBF requires better understanding of political economy issues – in particular how RBF is elevated and sustained in the policy agenda, how stakeholder interests are framed, and how opportunities are seized to advance RBF [6]. Deliberations among stakeholders, both those opposed to and supportive of RBF approaches, are all useful in expressing interests, understanding core concerns, and enabling collaboration to occur within policy development processes [8,11,12].

A number of RBF initiatives have been implemented as stand-alone projects, or pilots, in Uganda, albeit with little integration of RBF into the national health system [10,13,14]. Various small-scale RBF-related dialogues have taken place in Uganda since the seminal pilot conducted by the World Bank Research Group in 2003–2007. These dialogues were often organised to disseminate evidence of their effectiveness in improving healthcare outputs such as maternal deliveries and outpatient presentations. The dissemination meetings typically involved pilot implementers (mainly from non-state-run agencies), pilot funders and representatives of beneficiary groups. As observed by Ssengooba et al. (2015), these elite dialogues had limited participation from the legitimate institutions of government or health sector officials until 2014–2015 when the policy momentum increased and dialogues broadened [15]. During this time, three national dialogues were convened [16,17] to discuss the Ugandan experience of implementing RBF pilots with experts from other countries.

During 2014–2015 the Ministry of Health (MOH) in Uganda championed efforts to develop a customised national RBF model – mostly to help align the diverse RBF designs and implementation arrangements with health sector financing frameworks and institutions [16,18,19]. The Health Sector Financing Strategy (2015–2020) [19] explicitly recommended the customisation and scale-up of RBF as part of the country’s national plan for health financing.

From 2003 when the World Bank Development Group, in collaboration with Uganda’s health sector, commissioned the first RBF pilot, there was no official expression of government interest with regard to the scale-up of RBF [20]. Reports published over several years expressed major reservations with respect to the adoption of RBF – citing complexity and sustainability as major challenges [6,7,11,21]. In this study, we ask: Why has there been a rapid progression of RBF within the policy agenda in Uganda during 2014 and 2015, after ten years of apparent indifference? In order to address this question, this paper examines plausible explanations (policy arguments) for the observed policy developments. We aim to contribute to the scale-up of RBF in Uganda and other countries by exploring the agenda-setting processes for the policy and its drivers.

## Conceptual framework: policy deliberations for scale-up decision-making

The conceptual framework was derived from the work of Sabatier [22] and Kingdon [23]. Together, the two theories provided insights, concepts and main elements for generating alternative explanations about the scale-up of the RBF in Uganda. We used Sabatier and Jenkins’ Theory of Advocacy Coalitions to explain the emergence of competing sub-groups with favourable and unfavourable beliefs about policy problems in healthcare financing. This approach also showed how these beliefs can help mobilise coalitions across government and non-government to advocate and influence RBF policy change. The theory also draws attention to the way competing sub-groups frame and push their own narrative and define the policy problem and solutions in line with their preferred beliefs and values regarding policy impacts and costs, and the means to achieve desired outcomes.

Kingdon’s Multiple Stream Theory was also used to help explore how problems, solutions and politics create windows of opportunity to propel RBF onto the policy agenda [23]. According to Kingdon’s Theory, solutions such as RBF ascend onto the policy agenda if the beliefs about the perceived impacts intersect with the priority policy problem (e.g. healthcare financing), thus enabling those in authority (i.e. politicians) to solve the problem [23]. Both theories recognise the role of policy entrepreneurs – as individuals or coalitions of advocates, that take the initiative to elevate issues like RBF onto the national policy agenda by creating narratives and actions for coupling the three streams. Together, the theories allowed the exploration of policy actions with regard to advocacy and influence, as well as the assessment of RBF feasibility from the perspective of policy decision-making.

We also sought to identify dominant narratives influencing the policy agenda to scale up RBF in Uganda from the perspective of Advocacy Coalition Theory [22]. Data about the interests and concerns regarding the scale-up of RBF was collected from RBF stakeholder organisations (coalitions), meetings and reports [16,17]. Using Multiple Streams Theory, we explored the opportunities, challenges and champions for RBF policy development processes by interviewing respondents from the RBF practice community and decision-makers in government and healthcare provision.

In order to explain why RBF rapidly moved up the health policy agenda in Uganda, we examined the discussions and framing of RBF-related issues, problems and solutions at national and sub-national levels within Uganda’s health system. Our analysis, and in particular the process of weaving together the policy arguments, was guided by the work of Stone (1989). According to Stone, the vital factors for weaving together plausible storylines or narratives are: 1) the centrality of the causal mechanism; 2) defining the policy problem; 3) matching a problem with a feasible solution; and 4) addressing the main contentions across these elements [22,24]. In this paper, we examine alternatives, or competing arguments, for and against the policy scale-up of RBF in Uganda.

## Methods

This case study explores and synthesises interests, drivers and narratives regarding the RBF scale-up in Uganda. Qualitative methods were used to collect and analyse information from RBF consultative meetings, reviews of RBF-related documents and key informant interviews. Guided by theory, we constructed the main elements in the deliberations – i.e. the framing of arguments and contentions that surround RBF scale-up processes in the Ugandan health sector. Although the study was triggered by events that occurred over the period 2014 to 2015, this analysis is iterative, drawing on both current and historical events in order to construct meaningful arguments and explanations for RBF scale-up.

### Participant observation

The authors were active participants during the stakeholder meetings and served as members of the RBF technical working group led by the MOH. We acknowledge that this research approach can bias observations. However, it also allows a synthesis of complex events and dynamics that characterise policy development processes [25]. Three national consultation workshops were held – the first, in March 2014, was organised by the World Health Organisation (WHO) and the MOH in Uganda [17]. The other two were organised by the MOH and the Belgian Development Agency [16] and conducted during the first half of 2015. These workshops provided opportunities to bring stakeholders together to discuss RBF.

### Document review

The authors accessed and analysed contemporary debates about RBF within formal platforms such as technical workshops and national consultative meetings of stakeholders, and through documents about RBF. Reports of special studies and evaluations of pilots and grant applications with RBF programming were included in the document review. Scientific publications, implementation-level and national documents pertaining to health financing and strategic purchasing were of particular interest. The reports were sourced from the implementers and funders of RBF pilots, online reports and studies about RBF in Uganda. Information was also obtained from the MOH and agencies involved in the RBF pilots. From the reports we extracted background information, justifications, challenges and experiences for or against using RBF. As presented by Ssennyonjo et al. (2021) in this *Special Issue of Global Health Action* [1], many of these documents outlined the achievements and challenges faced while implementing RBF in Uganda’s health system. The documents that covered pilot studies were often framed from the perspective of implementers reporting the effectiveness of the pilots to funders, and making recommendations for government action to scale up RBF [20].

### Stakeholder interviews

During the study period (November 2014 to April 2015), a total of 39 interviews were conducted with stakeholders. These key informants were mainly individuals who actively participated in the design, financing, implementation and evaluation of the RBF schemes. Key actors in the national RBF pilots and programmes were identified from the literature and national consultative meetings. The categories of respondents covered implementers of RBF schemes (10), health facility managers (7), national-level decision-makers (from Government Ministries, academic and private sectors) (10), district health managers [7] and development partners (donors to RBF schemes) (7). Although the main study covered other issues related to RBF, such as the slow evolution of RBF in Uganda, this paper is based on questions related to the sudden interest in the policy to scale up RBF at the time of study.

Participants were interviewed about their organisations’ interests and the interests of other agencies in the scale-up of RBF in the health sector in Uganda. These question included: 1) the role they played in RBF; 2) realised benefits; 3) the extent to which RBF has moved up the policy agenda; and 4) in their view, the main reasons for the evolution and scale-up of RBF. We also explored questions regarding the fit between RBF and the institutions responsible for financing, health system performance management, opportunities, challenges and the champions for RBF policy development processes. The questions were informed by the Advocacy Coalition and Multiple Streams theories that guided the analysis in this paper. The interviews lasted between 40 and 60 minutes.

The study received ethical clearance from both Makerere University School of Public Health and Uganda National Council of Science and Technology. Informed consent was sought from participants before interviews were conducted. Participants in meetings were informed about the study and were assured of their anonymity in the study recordings and analysis and reporting.

### Analysis: the construction of arguments

This paper presents the main arguments which explain why RBF has made progress within Uganda’s policy agenda. To identify relevant findings, our analysis followed the elements expected in the framing of policy arguments as advised by Stone (1989) [24]. These elements include clarifying the causal links and contentions that explain the persistence of the policy problem, its mechanism and context. This analytical approach is also supported by Pawson and Tilly [26], who recommend a combination of data (both macro and micro) from both individuals and institutions with the aim of building explanatory completeness, synthesis or closure. The arguments were iteratively refined by re-examining and triangulating data from meetings and documents. We reviewed the interview data as the main arguments emerged in order to search for confirming or disconfirming narratives linking RBF to the beliefs and expectations of the respondents [27]. Beliefs and values were described as desirable and undesirable outcomes arising from RBF and its effects on healthcare. Examples included better performance, financial risk protection and service coverage.

At the centre of the analysis was the identification of core expectations (beliefs and values) arising from RBF and its scale-up in Uganda by individuals and organisations (coalitions).The main themes were related to how problems and solutions to RBF were defined. Other themes related to the contextual factors for RBF’s policy agenda, the opportunities and challenges that were being encountered while implementing RBF schemes, and aspirations and contestation about RBF scaled-up.

## Results

The study identified four plausible reasons, or policy arguments, for the advancement of RBF in Uganda during the study period. These are presented here as separate policy arguments, although there are some overlaps. They are summarised as: 1) the influence of external funding; 2) the need for cultivating national ownership of RBF implementation; 3) the development of RBF-related capabilities, also referred to as the ‘accumulation of expertise argument’; and 4) contentions against RBF scale-up – presented here as the arguments about ‘health systems challenges’ – mostly covering fundamental inadequacies such as funding, workforce and medicines. These are described below.

### Influence of external funding

Interviews from donor agencies and recipients of donor funds for implementing RBF schemes mostly framed RBF as a solution to address the problem of sub-optimal ‘value-for-money’ in the financing of the health programmes. ‘Improving efficiency, curbing corruption’ and ‘holding providers to account’ were common expressions in the interview scripts from donor agencies and their recipients. The wastage of funds through corruption and weak accountability for results were prevalent in the reports and interview narratives as the main elements in the framing of the policy problem to which RBF was proposed as a solution. RBF was also viewed as the main innovation for active performance management – linking donor financing directly to Millennium Development Goals (MDGs) targets. For this category of respondents, RBF was viewed as an innovation that works well to improve the results and efficiency of public health programmes. The main context of the narrative for this argument was the slow progression towards the MDGs in Uganda at the time and the perceived leakage of donor funds.

It [RBF] also checks on corruption a lot. That’s why people are embracing RBF [… …] it encourages transparency. If RBF is the one now to transform the health system, donors would rather go in for it. (RBF implementer)

I think MDGs have had an influence, especially within the last five years when there was that push to accelerate progress towards achieving the MDGs. They really made a case for RBF. […]. So MDG influence has really played a big role [in pushing for RBF] particularly in the last 5 years. (RBF donor/fund holder)

Narratives from interviews with donor agencies highlighted a reduction in donor funds available to support health programmes in countries like Uganda, and the urgent need to focus on results and their visibility to taxpayers abroad. RBF was seen as a useful tool to achieve these dual objectives. External funders also framed RBF as an evidence-based innovation, with Rwanda commonly being cited by respondents as a ‘success story’ for RBF scale-up in the region. Preconditions for donor financing – commonly cited as ‘conditionalities’ – were rife among respondents. The document review also provided proof that many donor funds had incorporated RBF as one of the preconditions for financial aid to health programmes in Uganda and beyond [28–31]. Many global aid providers had championed the RBF practices in global health programmes. The World Bank, the Global Alliance for Vaccine Initiative (GAVI), the Global Fund and the Presidential Emergency Programme for Air Relief (PEPFAR) had set service targets and applied varying degrees of RBF in their programmes. International private enterprises had seized the opportunity to play major roles and benefit from business opportunities linked to RBF programmes.

[…] the fact that the resource envelope globally is also getting smaller, there is an increased push for results. The taxpayers (in America and Europe) are asking their governments whether there is value for money in the investments. They ask: ‘Where are you investing our money? Can you demonstrate the impact of our intervention?’ So [the] increased push for results is a global trend due to increased scrutiny for ‘value for money’. (RBF donor/fund holder)

The motivation to adopt RBF is based on analysis of aid effectiveness strategies and it is widely acknowledged that handing out grants for which you seek only financial accountability is not necessarily the best way for achieving development outcomes. […] So RBF is in response to some of the dilemmas that donors face. (RBF implementer)

RBF has brought many non-medical organisations, whether you want to call them entrepreneurs, to the health sector. You will hear PricewaterhouseCoopers and Crown agents are now key players in RBF. You hear Marie Stopes is getting billions (shillings) to distribute RBF vouchers. […] There are business opportunities that RBF opened up at many levels […] to benefit from (funds for) health programmes. (Academic expert)

### National ownership of RBF

Although many RBF pilots were implemented in Uganda before 2015, most interviews and RBF engagements identified a problem – the non-involvement of government, the MOH and public providers – as part of the main policy problem hindering RBF scale-up. There was concern that the non-governmental agencies had over-extended their roles in RBF in a way that marginalised legitimate institutions in government. Most pilots worked with private providers and with variable involvement of district local governments and ignored the public-sector providers that constitute the majority of service providers in poor communities. Lack of autonomous financial management systems was frequently cited in the documents as a reason for the exclusion of public providers from RBF schemes.

During the study period (February 2014 to November 2015), steps were taken by Uganda’s MOH to actively engage and steer RBF. At the time of this study, the MOH had established an RBF Unit within the MOH headquarters to guide the design and harmonisation of RBF processes across the health sector. The unit had commissioned studies aimed at customising RBF design for Uganda’s health system and its institutional arrangements. The RBF Unit had also commissioned training programmes to orient both national and district health managers. This further demonstrates the process of integration of RBF within MOH structures. The anticipation of more donor funds being oriented towards RBF was another reason, according to respondents, for the increased involvement and interest of the MOH in directly steering the RBF developments during the study period.

As observed during RBF consultation meetings, district leaders were increasingly engaging the MOH to have their districts involved in RBF schemes – partly as a way of securing access to the funds that the government might obtain from RBF-inclined donors. For example, the government negotiated an RBF-conditioned loan for maternal and neonatal health from the World Bank at the time [32]. This realignment between district authorities and the central government to implement RBF programmes added credence to the government ownership argument.

Some schemes have been implemented as vertical stand-alone projects and not integrated [into existing systems]”. (…). “Patchy pilots all over the country make it difficult to scale up. (RBF donor/fund holder)

RBF schemes have moved slowly from external agencies such as Cordaid, Marie Stopes and others, to using district officials and national agencies with the rightful mandate for financing and provision of health services. (Academic expert)

PBF has caught attention of the MoH and many donors. There is ongoing synthesis of evidence and recently, even discussions of one RBF model. (MOH official)

The national ownership argument also included elements related to operations research to support RBF programmes by the academic institutions. The document review and interviews revealed that local researchers were actively involved in discussions and technical meetings in which the government was being advised on how to customise RBF models to fit the institutional set-up of the health system in Uganda [21,33].

Academic institutions are important in designing robust designs to generate localised evidence. This is important to demonstrate that RBF works or not. (Academic expert)

The national ownership argument had some disconfirming views among the stakeholders interviewed. First, a number of respondents observed the limited participation of key government agencies outside the MOH. A few respondents pointed out the lack of engagement of powerful stakeholders such as the Ministry of Finance, the Ministry of Local Government and the National Parliament, which are central to the operationalisation of public finance rules. These bodies hold legitimate roles that affect how RBF is implemented.

Those who have the money in Finance (ministry), the politicians have not understood it … and that is why they are dragging their feet for the scale-up process. (RBF implementer)

Those with power have no information; those with information have no power. How do you get a policy of RBF for only one sector (health) and leave out other government sectors? Rarely do government make policies by exception. RBF is about performance issues that cut across all government entities. […] Successful RBF policy requires broad engagement across government. (Academic expert)

Second, although this ownership argument is consistent with government-wide efforts to improve performance in other service sectors (28), the specification of service targets and auditing of results as required by RBF had not become operational outside the health sector. For instance, the idea of subjecting top-level government officials to ‘performance-based contracts’ was explicitly stated in the government’s Second National Development Plan launched in 2015. ‘Result-oriented thinking’ was being introduced in government budgeting frameworks as an ‘output-based budgeting tool’ [34] at the time of the study. These disconfirming views indicate that the ownership argument for the scale-up of RBF policy is limited to the health sector. This may cause challenges and confusion if it is not enshrined within a government-wide policy process.

This [Output Budgeting Tool] is not really performance-based initiative as they don’t link performance to rewards. There are no mechanisms to utilise the information generated. They are mimicking RBF but poorly. (MOH official)

We can see attempts on the part of the government to begin to think about results with the output-based budgeting they are implementing. They are talking about the performance-based budgeting. You can see in some sectors they are doing the performance contracts. These are attempt to begin holding people accountable for the funds they are receiving. (RBF donor/fund holder)

### Accumulation of RBF expertise

The third plausible explanation related to what was variously referred to as the accumulation of knowledge, capacity and experience with regard to RBF implementation. This was described by technocratic stakeholders as elements of RBF that had demonstrated effectiveness.

Interview scripts coded under this theme mostly related to increased expertise, especially among district-level actors – district health officials, non-government providers and pilot operatives. Respondents who had previously participated in the implementation of RBF schemes were confident of their capacity to scale up RBF schemes. In the districts that benefited from exposure to RBF, practical experience and capacity to support RBF operations were referred to as ‘adequate’, ‘optimal’ or ‘excellent’. Ministry-level officials also indicated that they too had increased their knowledge for RBF. The narratives about the historical evolution of the RBF pilots as well as the document reviews, provided a somewhat paradoxical view. Foreign experts and international non-governmental organisations all provided technical assistance for stewardship for the RBF programmes . The implication is that there was little opportunity for local expertise to develop, and a perception among policymakers that RBF was not a feasible or sustainable innovation, especially because of inadequate expertise within the MOH. As one respondent explained, ‘*Building local expertise was valued usually at the tail-end of the PBF pilots as the exit strategy. This elevated the focus on sustainability of the pilots by government at the time grants were drying out*.’ The involvement of the MOH officials at the dissemination stage of the pilot rather than during the design and implementation stages limited their exposure to RBF. In the experience of the first author, many of the questions that MOH officials would have asked in the inception phase of the pilot were being asked at the end of the pilot. This implied that there was more to be done to build the expertise among government officials to steer RBF.

Nonetheless, it was clear from the interviews that the RBF pilots in Uganda had increased interest, especially among district-level implementers. Many interview narratives indicated that lessons from prior pilots were being used to inform the design and implementation of newer RBF schemes over the years. The MOH was able to tap into this rich experience when it commissioned a study to examine the best model to recommend for national scale-up efforts in 2015. As one of the MOH officials stated, *‘The* g*enerated evidence from other schemes is now being used to inform the design of pilot by BTC and the national RBF model being proposed by MOH*.’

At MOH-level, several officials were becoming exposed to the operations of RBF within Uganda and other countries. Many officials had been sponsored to visit Rwanda and attended regional and international meetings on RBF for the period 2013–2015. These visits were aimed at supporting policy learning among MOH officials and encouraging them to push the national policy agenda on RBF in Uganda. The experiences of national RBF schemes in Rwanda, Burundi, Argentina and Zimbabwe as well as the RBF pilot projects in Uganda were shared in national RBF dialogues.

MoH has tried to bring different people who have done different schemes under one roof to share experiences. (MoH official)

[I am] aware of other schemes; we got to know them when WHO called us together as mobilisers. (RBF implementer)

I know Rwanda has done very well [with RBF […] As you’re aware, the MoH has tried so many tools to improve the quality of health services but many of them are not working and of late people think that RBF can make a difference. (RBF donor/fund holder)

In general, respondents exhibited enhanced understanding and appreciation of the operational feasibility and challenges of implementing RBF schemes. Nonetheless, policy-level respondents expressed a need for more information about RBF, especially regarding the costs.

### The health systems challenge

This argument held mixed perspectives, especially in terms of the prevailing health system constraints on the operational feasibility and sustainability of the RBF agenda in Uganda. Most respondents at the operational level (i.e. district officials and facility managers) pointed out several benefits of RBF but presented long lists of constraints on its operational feasibility.

Some respondents agreed that RBF schemes can strengthen public sector management and health system performance. For RBF schemes that distributed vouchers at the community level, improvements in the health-seeking behaviours were also observed as benefits. Likewise, benefits were reported among a few schemes that provided incentives for transport providers to take pregnant women to the health facilities.

[The Scheme] made it easy to access the facilities by the mothers so the mothers were saved from walking long distances. It was the responsibility of the transporters to look for the mothers [to take to the facilities] as they were also gaining. (RBF implementers)

The narratives show that bonuses – the main form of RBF payments – were important in motivating staff at health facilities to be more productive, despite the frequent concern in the interview data that payments of rewards to staff had low priority. In many cases, RBF funds were used to address shortages in medical supplies and medicines, and to pay for utilities such as water and electricity. Renovations of infrastructure using RBF bonuses were frequently reported at several healthcare facilities.

RBF bonuses should not be all spent at facility level … not even on staff parties (cerebrations). To appreciate RBF, people should feel that the money makes a difference in their pockets and their lives. (RBF implementers)

Numerous documents and respondent narratives confirmed the financial shortfalls in financing the inputs required to address RBF and non-RBF service targets. For example, concerns about the shortage of health workers and widespread stock-outs of medicines were said to offset the benefits of RBF. Interview narratives also indicated that the cost and sustainability of RBF programmes were not well addressed within the design of RBF schemes and the information shared with government officials. As one official from the planning department of the MOH put it, ‘*Research institutions like [… .] should assess PBF costs and sustainability. Information about sustainability has not been optimal*.’ Similar narratives showed concern over expensive RBF designs and poor considerations for sustainability after the donors withdraw. Overall, the health systems challenges indicate that the scale-up of RBF remains vulnerable to the resource constraints in healthcare provision.

## Discussion

This study investigated plausible explanations for the increased attention given to RBF in Uganda’s health policy agenda. In so doing, we sought to identify the drivers and the remaining challenges for this policy agenda and discourse. During 2014–2015, a new Health Sector Development Plan was developed [35] for the subsequent five years. The plan explicitly called for the application of RBF in the financing of health sector programmes. The RBF Unit was created within the MOH to coordinate RBF-related developments within the health sector. With the support of donor agencies, this unit successfully commissioned RBF studies and championed the development of a customised RBF model to fit the institutional set-up in the Ugandan health system [1]. At the time of publishing this paper, the government had rolled out the RBF project in 78 (out of 128) districts for maternal and neonatal services [36]. These developments are testament to the rapid scale-up of the RBF policy and programmes in Uganda’s health sector.

We present four overlapping plausible explanations as to why these developments gained policy momentum during the period 2014 to 2015. They point to: 1) the role of external aid; 2) the need to build national ownership, 3) increasing capabilities for implementation and stewardship of RBF programmes; and 4) diverse levers for and constraints on sustainable health systems performance. Together these explanations reveal issues at the centre of the policy agenda for RBF. The challenges of sustaining RBF on the policy agenda and the efforts needed to galvanise decisions for its application at the national scale are also evident.

The external funding argument was the strongest in shifting the RBF policy agenda in Uganda. Over 60% of Uganda’s health programmes are financed through development assistance from external donors [30]. The inclusion of RBF as a pre-condition in donor financed programmes has expanded since the MDGs era [6,37]. The proliferation of RBF-oriented global health financing initiatives geared at supporting countries like Uganda represents a window of opportunity for RBF. Making RBF one of the eligibility criteria for accessing external aid funds has provided the incentive for Uganda’s technical experts to prepare for the policy for RBF scale-up. The prospects for this financing and the influence of funders like the World Bank contributed to the elevation of RBF on the health policy agenda in Uganda. Other major donors with RBF programming included the Global Fund, USAID, UK-Aid and Belgium Technical Cooperation [1]. However, this also risks symbolic policy development – i.e. making policy for situational convenience or contingency in contrast to policy commitment to solve a well-articulated public problem over the long term [23,38]. The transition from external aid to more stable financing alternatives at the national level highlights the continuing policy dilemma for sustainability and scale-up of health financing innovations like RBF, especially when domestic revenues are limited [31,39,40]. Experience shows that countries that have integrated external aid revenues within the national RBF model have experienced major difficulties when external aid declines [41].

The national ownership argument in this study captured efforts to customise the RBF design features – from the first generation of pilots that were well-funded, expert-driven and mostly targeted to the private sector providers. This argument also takes into account a common pattern – where governments take the back seat in the RBF design and implementation –, making the pilots less responsive to capabilities and the needs of the public health system that the majority of poor people use [29,37].

The next generation models of RBF in Uganda have attempted to align with the decentralised institutional arrangements for service provision. This argument illustrates a search for cost control, feasible design and sustainability by optimising the fit of RBF within the health system and the institutional set-up for both the public and private health sectors in Uganda. Suffice it to note that the RBF models piloted before 2010 were mostly based on a rigid model advocated by the World Bank Development Group. Following the evaluation of the RBF schemes supported by the World Bank, Brenzel (2009) recommended the customisation of these schemes and more engagement of country-level stakeholders and capacity-building as a way of encouraging national level sustainability [42].

The past neglect and circumvention of the public sector by nearly all first-generation RBF pilots was perceived as a key factor that slowed down the policy development process [1]. From this perspective, RBF policy entrepreneurs need to engage directly with both the public and private sectors for a more expedient policy process. Lack of autonomy in decision-making about finances, workforce deployment and insufficient healthcare inputs were commonly cited as reasons for excluding the public sector from RBF schemes [7,21,43]. Where RBF schemes have been scaled up, engagement in solving these problems and strengthening health systems were crucial [2,4,5,8,9].

Our findings show that bypassing public sector providers is unlikely to advance the concerns of national ownership of the RBF policies and implementation arrangements. The dominance of public provision of health services in Uganda calls for RBF models that can engage and optimise public sector participation as well as help to solve the persistent shortages of healthcare inputs. The limitation of autonomy notwithstanding, the RBF models need to build capacity across public and private provider systems. This finding differs from the hands-off approach advocated by some commentators [3] who believe in unhindered innovation in the private sector as the main source of performance improvement.

The knowledge accumulation argument for RBF policy development relates to the capability development of legitimate institutions to implement RBF programmes. This explanation was closely related to the national ownership argument. Expatriates and international non-governmental organisations dominated initial periods of RBF implementation in Uganda and similar countries – thus portraying RBF as a heavily technical intervention that required outsourcing to highly capable agencies from Europe and the USA. From this perspective, the benefits of repeated piloting in different parts of the country and for different health programmes expanded opportunities for learning and more local expertise, especially among district officials and facility managers.

Academic expertise and operations research about RBF were also established in Ugandan universities. Home-grown academia with a focus on RBF was viewed as being crucial to the policy developments in the country. Although not prominent in the study narratives, the prospect of a national social health insurance scheme was an important contextual factor in the desire to scale up RBF [44]. This discourse was mostly concerned with preparing the health system for key capabilities such as strategic purchasing, provider payment systems and elaborating the health benefit package – all central to the successful launch of the insurance scheme.

### Perceived constraints on RBF scale-up

Despite the above three forward-looking arguments to scale up RBF and several-generation pilot designs and improvements [1], the arguments regarding constraints on RBF scale-up articulated a set of both structural and operational barriers. Among other factors, weak integration of RBF within the public finance system and a narrow focus on the health sector were viewed as insufficient for sustainable policy change. Government-wide policy development was viewed as essential to avoid ‘health sector exceptionalism’ that may meet resistance if government-wide rules remain unchanged (29). Operationalising RBF for teachers, the police, the prisons service, the army and other service sectors in Uganda would help to address RBF as a government-wide policy. Insufficient government healthcare financing, especially in the public system, was a major constraint in Uganda and similar countries [5–7].

Some respondents were concerned about the high costs of outsourcing administrative functions for RBF to non-state agencies instead of existing institutions with these mandates. These practices were viewed by some respondents as ‘business schemes’ that were diverting funds away from direct service provision. By extension, the information on RBF costs remains less publicised in Uganda and from the global literature.

### Study limitations

We acknowledge that the study has some limitations. Including only a limited number of potential explanations may have excluded some prominent perspectives and viewpoints. All authors have had some involvement in the implementation and evaluation of Uganda’s RBF pilots in the last seven to ten years. However, we considered that participation by the first author (FS) was useful to appreciate the complexity of the RBF journey. The mix of academic and policy practice experts helped to balance the discussions and internal reflections. The study did not integrate the perspectives of global actors. We acknowledge that global actors are likely to influence the RBF design and programme objectives in countries such as Uganda.

## Conclusion

The different explanations for the ascendance of RBF onto the national agenda for policymaking in Uganda illustrate the main concerns and potential solutions to RBF in Uganda and similar countries. Although donor-aid preconditions and the coupling of aid to results are arguably the most potent factors shifting the policy agenda in favour of RBF scale-up, building domestic capacity to implement RBF within the legitimate institutions of central and local government is imperative.

## Data Availability

Interviews are available within data sharing protocols at Makerere University School of Public Health. https://sph.mak.ac.ug.

## Paper context

Innovations to finance programmes to address the goal of universal health coverage have advocated the use of the results-based financing. This study explores the forces that have elevated results-based financing in Uganda’s policy agenda. The external aid argument was found to be the strongest reason. If results-based financing is to find a good fit in the health system in Uganda and similar countries, policy advocates should clearly articulate the need to: 1) enshrine RBF in the demands for improved domestic financing for healthcare; 2) reduce the associated administrative costs; 3) ameliorate the widespread shortages of inputs such as the workforce and medicines; and 4) broaden advocacy and engagement beyond the health sector.

## Data deposition

Not applicable.
